# SYMPK Is Required for Meiosis and Involved in Alternative Splicing in Male Germ Cells

**DOI:** 10.3389/fcell.2021.715733

**Published:** 2021-08-09

**Authors:** Rui Wu, Junfeng Zhan, Bo Zheng, Zhen Chen, Jianbo Li, Changrong Li, Rong Liu, Xinhua Zhang, Xiaoyan Huang, Mengcheng Luo

**Affiliations:** ^1^Department of Tissue and Embryology, School of Basic Medical Sciences, Wuhan University, Wuhan, China; ^2^Hubei Provincial Key Laboratory of Developmentally Originated Disease, Wuhan, China; ^3^Reproductive Medicine Center, Department of Obstetrics and Gynecology, Affiliated Hospital of Guizhou Medical University, Guiyang, China; ^4^Department of Urology, Zhongnan Hospital of Wuhan University, Wuhan, China; ^5^Center for Reproduction and Genetics, Suzhou Municipal Hospital, The Affiliated Suzhou Hospital of Nanjing Medical University, Suzhou, China; ^6^State Key Laboratory of Reproductive Medicine, Department of Histology and Embryology, Nanjing Medical University, Nanjing, China

**Keywords:** meiosis, SYMPK, alternative splicing, spermatocytes, meiotic recombination

## Abstract

SYMPK is a scaffold protein that supports polyadenylation machinery assembly on nascent transcripts and is also involved in alternative splicing in some mammalian somatic cells. However, the role of SYMPK in germ cells remains unknown. Here, we report that SYMPK is highly expressed in male germ cells, and germ cell-specific knockout (cKO) of *Sympk* in mouse leads to male infertility. *Sympk* cKO^Ddx4–cre^ mice showed reduced spermatogonia at P4 and almost no germ cells at P18. *Sympk* cKO^Stra8–Cre^ spermatocytes exhibit defects in homologous chromosome synapsis, DNA double-strand break (DSB) repair, and meiotic recombination. RNA-Seq analyses reveal that SYMPK is associated with alternative splicing, besides regulating the expressions of many genes in spermatogenic cells. Importantly, *Sympk* deletion results in abnormal alternative splicing and a decreased expression of *Sun1*. Taken together, our results demonstrate that SYMPK is pivotal for meiotic progression by regulating pre-mRNA alternative splicing in male germ cells.

## Introduction

Mammalian spermatogenesis is a continuous, well-organized process of germ cell differentiation including mitosis in spermatogonia, meiosis in spermatocyte, and spermiogenesis (Griswold, 2016). During this process, the gene expression pattern is highly dynamic and under precise control, which owes partly to alternative splicing (AS) and the polyadenylation modification of messenger RNAs (mRNAs) (Kashiwabara et al., 2002; Kan et al., 2005; Goldstrohm and Wickens, 2008; Weill et al., 2012). Additionally, several regulators of pre-mRNA splicing are primarily or specifically expressed in the testis (Schmid et al., 2013).

Previous studies have shown that there were abundant AS variants in spermatogenic cells (Yeo et al., 2004; Schmid et al., 2013; Margolin et al., 2014). Of note is that exon–exon junction (EEJ) and intron retention (IR) are the major AS modes in male germ cells (Chen et al., 2018). Recently, increasing evidences support the idea that AS plays an important role in mouse spermatogenesis. For example, PTBP2, which is required for the AS regulation of germ cell mRNAs, is critical for male germ cell survival and fertility (Zagore et al., 2015; Hannigan et al., 2017). In addition, many other RNA-binding proteins, such as RBM5 (O’Bryan et al., 2013), RANBP9 (Bao et al., 2014), BCAS2 (Liu et al., 2017), and the m6A reader YTHDC1 (Kasowitz et al., 2018), are also involved in modulating AS during meiosis and are indispensable for germ cell survival and mouse fertility.

SYMPK was initially identified as a protein associated with the tight junctions of polarized epithelial cells (Keon et al., 1996). The interaction of SYMPK with cleavage and polyadenylation and specificity factors (CPSFs) and cleavage stimulation factor F (CSTF) suggests its function in the assembly of the polyadenylation machinery (Takagaki and Manley, 2000; Hofmann et al., 2002). Further study confirmed that SYMPK is indeed necessary for nuclear polyadenylation (Xing et al., 2004) and cytoplasmic polyadenylation element-binding protein (CPEB)-mediated cytoplasmic polyadenylation in *Xenopus* oocytes (Barnard et al., 2004). In tumor cells, SYMPK is required for mitotic fidelity by supporting microtubule dynamics (Cappell et al., 2010). Importantly, SYMPK has been identified as a cofactor of RBFOX2 and NOVA2 to regulate alternative mRNA splicing (Misra et al., 2015). Recently, it has been reported that SYMPK promotes the self-renewal and pluripotency of embryonic stem cells (ESCs) through interaction with OCT4 (Yu et al., 2019). Nevertheless, the function of SYMPK in male germ cells is still unclear.

Here, we report that SYMPK is highly expressed in the testes and is indispensable for meiosis progression and mouse fertility. Disruption of *Sympk* leads to the dysregulation of transcription and AS in spermatogenic cells. Our data reveal the essential role of SYMPK in meiosis and male fertility.

## Results

### SYMPK Is Highly Expressed in Mouse Testes

**FIGURE 1:**
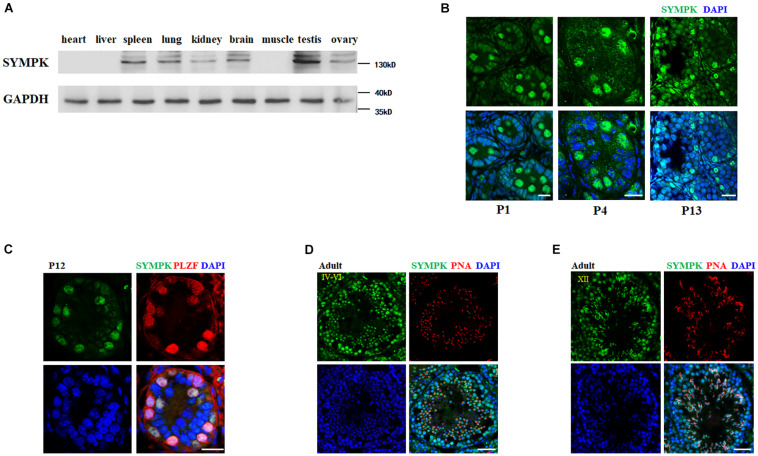
SYMPK is abundantly expressed in mouse testes. **(A)** Immunoblot analysis of SYMPK expression in different tissues from adult mouse. GAPDH was used as the loading control. **(B)** Immunostaining of SYMPK in mouse testes at postnatal day 1 (P1), P4, and P13. *Scale bar*, 20 μm. **(C)** Frozen sections of P12 testes were co-stained with rabbit anti-SYMPK and mouse anti-PLZF antibodies. DNA was stained with DAPI. *Scale bar*, 50 μm. **(D,E)** Immunofluorescence analysis of SYMPK in adult testes. SYMPK (*green*) and acrosome marked by PNA (*red*). *Scale bar*, 50 μm.

To explore the potential function of SYMPK in mouse spermatogenesis, we firstly determined the expression level of SYMPK in different mouse tissues by immunoblotting with an anti-SYMPK antibody. SYMPK abundance was much higher in the testes compared with other tissues (Figure 1A). Immunostaining was then used to determine the subcellular localization of SYMPK in mouse testes. In newborn mouse testes (postnatal day 1, P1), SYMPK was highly expressed in the prospermatogonia located in the middle of the seminiferous tubules (Figure 1B). At P4 and P13, SYMPK expression was relatively high in a subset of cells located close to the basement membrane, which are presumably spermatogonia (Figure 1B). Co-immunostaining of SYMPK and the spermatogonia-specific transcription factor PLZF confirmed that SYMPK was highly enriched in spermatogonia in P12 testes (Figure 1C). In adult testes, SYMPK was abundantly enriched in spermatogonia, spermatocytes, round spermatids, and elongated spermatids in stages IV–VI and XII of seminiferous tubules (Figures 1D,E). These data suggest that SYMPK probably plays important roles in the entire process of spermatogenesis.

### SYMPK Is Essential for Male Fertility

**FIGURE 2:**
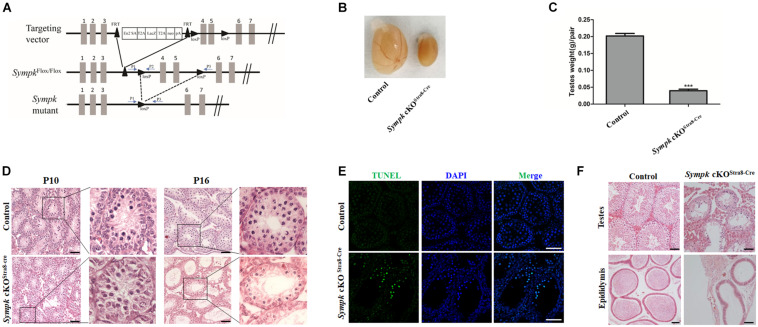
SYMPK is essential for male fertility. **(A)** Schematic diagram of the targeting strategy for the generation of *Sympk*^*F/F*^ and *Sympk* cell-specific knockout (cKO) mice. Exons 4 and 5 were flanked by loxP and will be deleted by Cre recombinase. The positions of the primers used for genotyping are shown as *arrows*. **(B,C)** Morphological analysis **(B)** and testis weight **(C)** of adult control and *Sympk* cKO^Stra8–Cre^ mice (***p < 0.001, *n* = 3). **(D)** Histology of testes from wild-type control and *Sympk* cKO^Stra8–Cre^ mice at P10 and P16. **(E)** Apoptosis of wild-type control and *Sympk* cKO^Stra8–Cre^ seminiferous tubules at P18 was analyzed with the TUNEL assay. *Scale bar*, 50 μm. **(F)** Histology of the testes and cauda epididymides from adult wild-type control and *Sympk* cKO^Stra8–Cre^ mice.

To investigate the physiological function of SYMPK in mouse fertility, *Sympk^*Flox*/+^* mice were obtained and used for generating the *Sympk*^*Flox/Flox*^ (*Sympk*^*F/F*^) mouse line, which allowed us to inactivate *Sympk* by deleting exons 4 and 5 in a cell- or tissue-specific manner using Cre recombinases (Figure 2A). It has been well established that Cre is active in the germ cells of *Ddx4*-Cre mice at embryonic day 15 (Gallardo et al., 2007) in the spermatogonia of *Stra8*-Cre mice at 3 days postpartum (Sadate-Ngatchou et al., 2008). The immunostaining experiments found that SYMPK was absent in germ cells from the testes of *Sympk* cKO^Ddx4–Cre^ and *Sympk* cKO^Stra8–Cre^ mice at P4 and P13, respectively (Supplementary Figure 1).

*Sympk* cell-specific knockout (cKO) male mice were viable and grossly normal. However, both the *Sympk* cKO^Ddx4–Cre^ and *Sympk* cKO^Stra8–Cre^ male mice were infertile. The testes of adult *Sympk* cKO^Stra8–Cre^ male mice were much smaller than those of the wild-type (*wt*) mice, with significantly reduced testis weight (Figures 2B,C). Similarly, testes from *Sympk* cKO^Ddx4–Cre^ males were also much smaller than those from *wt* mice (Supplementary Figure 2A). Histological analysis revealed that there was no apparent difference in the germ cell numbers in the seminiferous tubules between the *wt* control and *Sympk* cKO^Stra8–Cre^ mice at P10 (Figure 2D), while the number of germ cells was sharply reduced in *Sympk* cKO^Ddx4–Cre^ testes as early as P10 (Supplementary Figure 2B). Noticeably, the number of spermatocytes in *Sympk* cKO^Stra8–Cre^ tubules was significantly reduced compared with those in *wt* mice at P16 (Figure 2D). By TdT-mediated dUTP nick-end labeling (TUNEL) assays, increasing *Sympk*-deficient spermatocytes were positive for TUNEL labeling, which suggests that they were undergoing apoptosis (Figure 2E). The testes from adult *Sympk* cKO^Stra8–Cre^ males (Figure 2F) lacked post-meiotic spermatids, and the most advanced germ cells were spermatocytes, suggesting meiotic defects. Expectedly, no sperm was observed in the epididymis from adult *Sympk* cKO^Stra8–Cre^ males (Figure 2F). The testes and epididymis from adult *Sympk* cKO^Ddx4–Cre^ males were completely devoid of germ cells and sperm, respectively (Supplementary Figure 2C). The above data demonstrate that SYMPK is required for male fertility.

To determine whether SYMPK deficiency would affect the survival of spermatogonia, testis sections were stained with an anti-PLZF antibody. We found that the number of PLZF-positive cells in *Sympk* cKO^Stra8–Cre^ testes was comparable to that in *wt* testes at P13 (Supplementary Figures 3A,B), which suggests that the deletion of *Sympk* after birth does not affect spermatogonial survival. However, the number of cells expressing SYCP3 in *Sympk* cKO^Stra8–Cre^ tubules was much less than that in *wt* testes at P13, and some Sympk cKO^Stra8–Cre^ tubules did not contain SYCP3-positive cells (Supplementary Figures 3C,D). DDX4 is a germ cell-specific marker. We stained the frozen sections of P4 and P18 testes with the DDX4 antibody and the numbers of DDX4-positive cells in each seminiferous tubule were counted. The results showed that the number of DDX4-positive cells sharply reduced in *Sympk* cKO^Stra8–Cre^ males at P18 (Supplementary Figure 3G). Interestingly, the number of DDX4-positive cells was reduced in *Sympk* cKO^Ddx4–Cre^ males at P4 and disappeared at P18 (Supplementary Figures 3E–G), which suggest that deletion of *Sympk* at the embryonic stage has a negative influence on the survival of spermatogonia. Taken together, these results suggest that SYMPK was required for spermatogonial survival and meiosis in spermatocytes.

### *Sympk* Deletion Causes Defects in Chromosome Synapsis and DSB Repair

**FIGURE 3:**
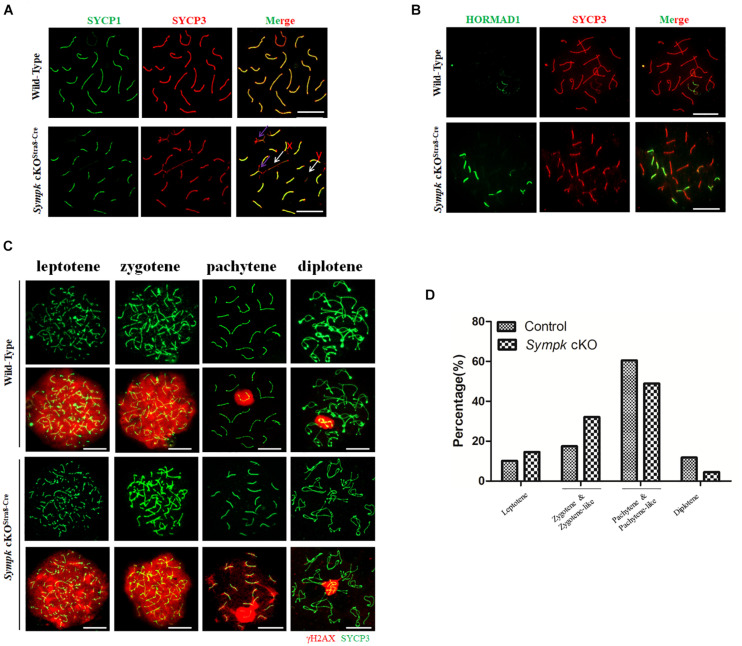
Failure of chromosome synapsis and double-strand break (DSB) repair in *Sympk* cKO^Stra8–Cre^ spermatocytes. **(A)** Chromosome spreads from wild-type (*wt*) control and *Sympk* cKO^Stra8–Cre^ testes were labeled for synaptonemal complex proteins (SYCP1 and SYCP3). *Purple arrow* shows the incomplete synapsis in the autosome of pachytene spermatocyte; *white arrow* shows the separation of the sex chromosome. **(B)** Spermatocytes from *wt* control and *Sympk* cKO^Stra8–Cre^ testes were immunostained for HORMAD1 and SYCP3. **(C)** Chromosome spreads from control and *Sympk* cKO^Stra8–Cre^ testes were immunostained for γH2AX and SYCP3. DNA was stained with DAPI. *Scale bar*, 20 μm. **(D)** Percentages of spermatocytes at different stages (leptotene to diplotene) from the testes of *wt* control and *Sympk* cKO^Stra8–Cre^ mice at P18. *wt* spermatocytes, 423; *Sympk* cKO spermatocytes, 339.

To further determine the role of SYMPK in meiosis, we tested whether *Sympk* deficiency affects chromosome synapsis and DNA double-strand break (DSB) repair, which are critical for meiosis progression. Homologous chromosomal synapsis was assessed by immunostaining of spread nuclei using antibodies against SYCP1, a component of the synaptonemal complex (SC) transverse elements, and SYCP3, a component of SC axial elements (de Vries et al., 2005; Kogo et al., 2012). Chromosomes from the *wt* pachytene spermatocytes maintained faithful synapsis, whereas many of the *Sympk* cKO^Stra8–Cre^ pachytene-like spermatocytes showed defects or failure of chromosome synapsis, as characterized by the absence of full autosome synapsis and dissociation of the sex chromosome (Figure 3A). HORMAD1, associated with unsynapsed chromosome axes (Fukuda et al., 2010), was also used to evaluate the chromosome synapsis. In wild-type pachytene spermatocytes, HORMAD1 only localized in the unsynapsed sex chromosomes, but in the *Sympk* cKO spermatocytes, several unsynapsed autosomes are positive for HORMAD1 (Figure 3B). The result from HORMAD1 staining further confirmed the synapsis defects in *Sympk* cKO spermatocytes.

DSB formation and its repair were assessed using the DSB marker γH2AX, which is phosphorylated by the ATM and ATR kinases (Bellani et al., 2005). The γH2AX signal was distributed similarly between *Sympk* cKO^Stra8–Cre^ and *wt* spermatocytes at the leptonema and zygonema, indicating that SYMPK is not essential for DSB formation (Figure 3C). During the pachynema and diplonema, the γH2AX signal was restricted to the XY body in *wt* spermatocytes, while in *Sympk* cKO^Stra8–Cre^ spermatocytes, γH2AX also localized in some autosomes besides the XY body (Figure 3C), indicating that DSBs were not properly repaired in *Sympk* cKO^Stra8–Cre^ spermatocytes at the pachytene and diplotene stages. In addition, XY body formation was impaired in some *Sympk*-deficient spermatocytes (Figure 3A). These results suggest that *Sympk* deletion leads to failure of the DSB repair. In addition, we quantified the spermatocytes at different stages from juvenile *wt* and *Sympk* cKO^Stra8–Cre^ males at P18. The results showed that fewer *Sympk* cKO^Stra8–Cre^ spermatocytes can progress into the pachynema and diplonema when compared with the *wt* males (Figure 3D), suggesting that *Sympk*-deficient spermatocytes were mainly arrested at the pachytene stage.

Previous studies have suggested that BRCA1 recruits the ATR kinase to phosphorylate H2AX on unsynapsed chromosomes (Turner et al., 2004) and has a role in repairing DSBs (Xu et al., 2003). Given the failure of chromosome synapsis and DSB repair, we wondered whether the localization of BRCA1 is also affected in *Sympk* cKO spermatocytes. Therefore, BRCA1 localization was determined and compared between the *wt* and *Sympk* cKO spermatocytes. At the zygotene stage, BRCA1 accumulated on the unsynapsed axes of chromosomes in both *wt* and *Sympk* cKO spermatocytes. At the pachytene stage, BRCA1 was restricted in the sex chromosomes in *wt* spermatocytes, but persisted in the unsynapsed chromosomes in *Sympk* cKO pachytene spermatocytes (Supplementary Figure 4). The localization of BRCA1 is consistent with the defects in DSB repair and chromosome synapsis in *Sympk* cKO spermatocytes.

### Deficiency of *Sympk* Impairs Meiotic Recombination and Crossover Formation

**FIGURE 4:**
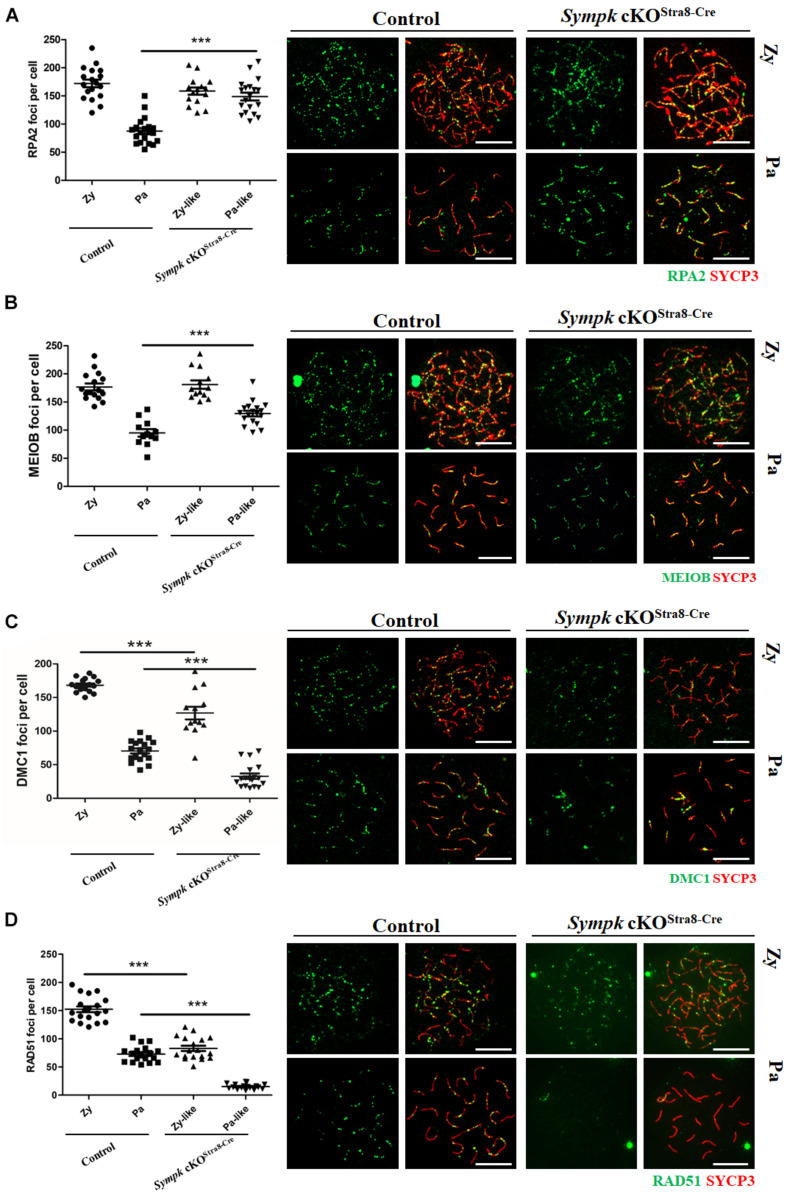
SYMPK is required for meiotic recombination. Chromosome spreads of spermatocytes from wild-type (*wt*) control and *Sympk* cKO^Stra8–Cre^ testes were labeled with RPA2, MEIOB, DMC1, RAD51, and SYCP3. Based on the staining pattern of the synaptonemal complex lateral element (SYCP3), the spermatocytes were classified into the following stages: leptotene (*Le*), zygotene (*Zy*), pachytene (*Pa*), zygotene-like (*Zy-like*), and pachytene-like (*Pa-like*). *Each dot* represents the number of DNA repair protein foci per cell. *Solid lines* show the average number of foci in each category of spermatocytes. Data were collected from three mice. **(A)** RPA2 foci. **(B)** MEIOB foci. **(C)** DMC1 foci. **(D)** RAD51 foci. Representative images of the spermatocytes at the zygotene, zygotene-like, pachytene, or pachytene-like stages are shown **(A–D)**. *Scale bar*, 20 μm.

To determine whether SYMPK deficiency affects meiotic recombination, the recombination nodules were evaluated by immunostaining with RPA2, MEIOB, RAD51, and DMC1 on spread nuclei. RPA2 is a subunit of the RPA complex that binds to single-strand NA (ssDNA) (Ribeiro et al., 2016). MEIOB, an ssDNA-binding protein, is one of the most important meiotic prophase I regulators that are required for meiotic recombination through interactions with SPATA22 and RPA2 (Luo et al., 2013; Lascarez-Lagunas et al., 2020). DMC1 and RAD51 are involved in DNA strand invasion and exchange (Brown and Bishop, 2014). Consistent with the defects of DSB repair in *Sympk* cKO^Stra8–Cre^ pachytene spermatocytes, the numbers of RPA2 and MEIOB foci increased in *Sympk* cKO^Stra8–Cre^ pachytene spermatocytes (Figures 4A,B). Meanwhile, the numbers of DMC1 and RAD51 foci decreased significantly in *Sympk* cKO^Stra8–Cre^ zygotene-like and pachytene-like spermatocytes (Figures 4C,D). The above aberrant recombination nodules suggest that meiotic recombination was defective in *Sympk* cKO spermatocytes.

In order to determine the effect of *Sympk* deficiency on crossover formation, the MLH1 foci were visualized on pachytene chromosomes. We found that the average number of MLH1 foci was 25.1/cell in *wt* pachytene spermatocytes (Supplementary Figures 5A,B), which is similar to that in a previous report (Anderson et al., 1999). In contrast, the number of MLH1 foci reduced to as low as four per cell in *Sympk* cKO^Stra8–Cre^ pachytene-like spermatocytes (Supplementary Figures 5A,B).

Taken together, our results demonstrated that, in the absence of SYMPK, the early steps of meiotic recombination, such as DSB formation and DNA resection, progressed well, while the subsequent steps, such as strand invasion, chromosome synapsis, DSB repair, and crossover formation, were impaired, finally leading to meiotic failure.

### SYMPK Interacts With DDX5 and PRPF8 in Spermatogenic Cells

**FIGURE 5:**
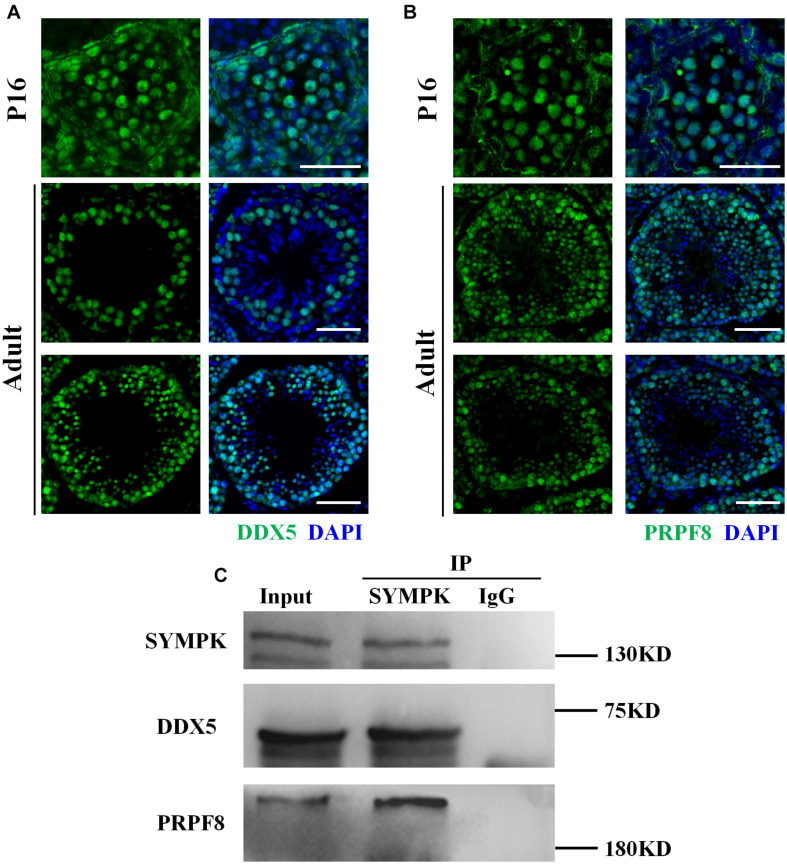
SYMPK interacts with DDX5 and PRPF8 in spermatogenic cells. **(A,B)** Localization of DDX5 **(A)** and PRPF8 **(B)** in P16 and adult wild-type testes. DNA was stained with DAPI. *Scale bar*, 50 μm. **(C)** Co-immunoprecipitation using the anti-SYMPK antibody or IgG confirmed that SYMPK interacts with DDX5 and PRPF8 in spermatogenic cells.

SYMPK has been previously reported to regulate the AS in somatic cells (Flp-In-293 cells) (Misra et al., 2015). Considering that the testes contain high levels of AS events (Elliott and Grellscheid, 2006), we asked whether the function of SYMPK regulating AS was conserved in germ cells. Firstly, we performed an immunoprecipitation–mass spectrometry (IP-MS) assay using an antibody against SYMPK to identify the splicing factors interacting with SYMPK in spermatogenic cells. Spermatogenic cells were enriched from *wt* and *Sympk* cKO^Stra8–Cre^ testes and used to determine the protein expression. Enriched spermatogenic cells were determined by Western blotting (Supplementary Figure 6). Multiple proteins associated with pre-mRNA processing were detected in the IP-MS results of the SYMPK antibody pull-down products, including pre-mRNA processing factors, i.e., PRPF8 (pre-mRNA-processing-splicing factor 8), PRPF19, PRPF40a, WTAP (pre-mRNA-splicing regulator WTAP), and CWC22 (pre-mRNA-splicing factor CWC22 homolog), and two RNA helicases, DDX5 [DEAD (Asp-Glu-Ala-Asp) box helicase 5] and DHX15 (pre-mRNA-splicing factor ATP-dependent RNA DEAH-box helicase 15). PRPF8 and DDX5 are two enriched putative SYMPK-binding proteins based on the number of matched peptides in the *wt* and *Sympk* cKO groups (Supplementary Table 3). These two proteins were chosen for further analysis. In both P16 and adult mouse testes, PRPF8 and DDX5 localized in the nucleus of spermatogenic cells, but not in elongated spermatids (Figures 5A,B). Due to the lack of suitable antibodies, the co-localization between PRPF8, DDX5, and SYMPK was not determined. Using *in vivo* co-immunoprecipitation assays, we confirmed that DDX5 and PRPF8 indeed interacted with SYMPK in spermatogenic cells (Figure 5C). Collectively, these results suggest a role of SYMPK regulating pre-mRNA splicing in spermatogenic cells.

### SYMPK Modulates Pre-mRNA Splicing in Spermatogenic Cells

**FIGURE 6:**
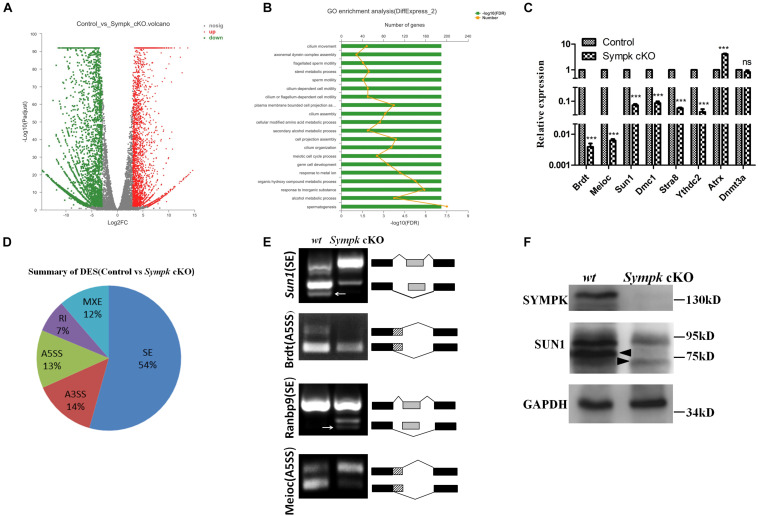
SYMPK is involved in transcript splicing during spermatogenesis. **(A)** Volcano plot of the differentially expressed transcripts in *Sympk* cKO^Stra8–Cre^ spermatogenic cells compared with the wild-type (*wt*) controls. *Green dots* represent significantly downregulated transcripts, *red dots* represent significantly upregulated transcripts (*Q* value < 0.05, fold change of RPKM > 2), and *gray dots* represent unchanged transcripts. **(B)** Gene Ontology (GO) term enrichment analysis of the significantly affected transcripts. **(C)** Validation of the differentially expressed genes by real-time PCR analysis. **(D)** Summary of five significantly affected alternative splicing (AS) events in *Sympk* cKO^Stra8–Cre^ spermatogenic cells at P18. **(E)** RT-PCR verification of the AS of transcripts in *wt* and *Sympk* cKO^Stra8–Cre^ spermatogenic cells. RT-PCR was performed with specific primers (Supplementary Table 1). *White arrow* stands for unspecific band. **(F)** Western blotting analysis of the expressions of *Sympk* and *Sun1* in *wt* and *Sympk* cKO^Stra8–Cre^ spermatogenic cells, with GAPDH as the loading control. The *solid triangle* stands for unspecific band.

To investigate the molecular consequences of *Sympk* deficiency in germ cells, whole-transcriptome RNA sequencing (RNA-Seq) analysis was performed using total RNA extracted from *wt* and *Sympk* cKO^Stra8–Cre^ spermatogenic cells. With a *p*_adjust_ < 0.01, a total of 6,528 transcripts showed a more than eightfold differential expression in SYMPK-deficient spermatogenic cells: within which, 2,651 genes were upregulated and 3,877 genes were downregulated (Figure 6A). Gene Ontology (GO) analysis identified that the spermatogenesis was most significantly affected (Figure 6B). Interestingly, only 10 meiotic genes were upregulated, while 72 genes associated with meiosis were downregulated. Reverse transcription quantitative PCR (RT-qPCR) results confirmed that six genes required for spermatogenesis were downregulated (Figure 6C). The dysregulated expressions of meiosis-specific genes may have contributed to the meiotic defects in SYMPK-deficient male germ cells.

To dissect the impacts of *Sympk* deletion on AS, the types of AS between the control and *Sympk* cKO spermatogenic cells were compared based on the reads on target and junction counts using the rMATS software. Compared with the control, 6,046 AS events were significantly affected (*p* < 0.01) in *Sympk* cKO^Stra8–Cre^ spermatogenic cells. Among the affected AS events, the majority of the changed splicing events were skipped exons (Figure 6D). Exon skipping is the most enriched AS form during mouse spermatogenesis (Schmid et al., 2013; Margolin et al., 2014). Moreover, the SYMPK-binding protein PRPF8 is a core component of spliceosomal complexes, which indicated that SYMPK participated in the AS regulation unbiasedly.

To validate whether AS is indeed disturbed due to SYMPK deficiency, four transcripts with alterations in the splicing pattern identified by rMATS were examined by semi-quantitative RT-PCR using specific primers (Supplementary Table 1), namely, *Sun1*, *Brdt*, *Meioc*, and *Ranbp9*. The predicted aberrant splicing patterns of these transcripts were successfully verified in the complementary DNA (cDNA) of *Sympk* cKO^Stra8–Cre^ spermatogenic cells (Figure 6E). These data demonstrated that SYMPK was involved in mRNA splicing during spermatogenesis.

### Depletion of *Sympk* Results in Decreases in SUN1 Protein

SUN1 is an inner nuclear membrane protein associated with telomeres during meiotic prophase I. Disruption of *Sun1* in mice impaired the efficient homolog pairing, synapsis formation, and recombination in meiosis (Ding et al., 2007). Interestingly, the AS pattern of *Sun1* is dramatically changed in *Sympk*-deficient spermatogenic cells (Figure 6E). The similar phenotype between *Sympk* cKO^Stra8–Cre^ and *Sun1* null mice promoted us to analyze the expression of SUN1 protein in *Sympk* cKO^Stra8–Cre^ spermatogenic cells. Compared with the control, the expression of *Sun1* was reduced dramatically in *Sympk* cKO^Stra8–Cre^ spermatogenic cells (Figure 6F). Thus, these results suggest that SYMPK deletion possibly decreased *Sun1* expression by changing its pre-mRNA splicing, subsequently leading to the meiotic defects.

## Discussion

Here, we report that SYMPK is essential for meiosis and male fertility. Although the deletion of *Sympk* in either embryonic or postnatal stage leads to infertility in male mice, development of the testes from mice with *Sympk* deletion at the embryonic and postnatal stages was different. In *Sympk* cKO^Ddx4–Cre^ male mice, the number of germ cells was reduced at P4 and disappeared at P18 in the testes. In *Sympk* cKO^Stra8–Cre^ tubules, we did not observe any reduction of spermatogonia, while the spermatocyte number was sharply reduced. Furthermore, *Sympk* cKO^Stra8–Cre^ spermatocytes displayed defects in meiosis progress, including chromosome asynapsis, DSB repair failure, defective meiotic recombination, and reduced crossover formation. Consistently, BRCA1 was localized to the unsynapsed chromosomes of *Sympk* cKO^Stra8–Cre^ spermatocytes at pachytene, not restricted to the sex chromosomes, which is different from that in *wt* spermatocytes. Considering the role of BRCA1 in recruiting the kinase ATR to phosphorylate H2AX on unsynapsed chromosomes and in meiotic sex chromosome inactivation (MSCI) establishment (Broering et al., 2014), the abnormal BRCA1 localization and defective meiotic DSB repair may have resulted in failure of meiotic silencing, finally causing the altered transcriptional profile and apoptosis of spermatocytes.

AS is the major posttranslational modification that is prevalent in the male germ cells and is particularly active in late pachytene spermatocytes and spermatids. Any mutation affecting AS would be harmful to successful spermatogenesis (Venables, 2002). Although SYMPK is not an RNA-binding protein, SYMPK and CPSF functioned as cofactors of two splicing regulators (RBFOX2 and NOVA2) to regulate AS in somatic cells. We wondered whether SYMPK would also participate in the AS regulation of germ cells. Here, we identified two SYMPK-binding partners (DDX5 and PRPF8) in spermatogenic cells through IP-MS. Similar to the localization pattern of SYMPK in the testes, both DDX5 and PRPF8 were localized in the nucleus of spermatogenic cells. In somatic cells, DDX5 coordinated with hnRNA1 to regulate alternative pre-mRNA splicing (Lee et al., 2018). Recently, it has been reported that DDX5 is required for the splicing of critical genes for spermatogenesis in spermatogonia (Legrand et al., 2019). PRPF8 plays a critical role in pre-mRNA splicing as the core component of spliceosomal complexes. In addition, PRPF8 also mediates the assembly of several spliceosomal proteins. It is reported that PRPF8 mutation caused aberrant splicing in some human diseases, such as myeloid malignancies and autosomal-dominant retinitis pigmentosa (Kurtovic-Kozaric et al., 2015; Xiao et al., 2020). Therefore, SYMPK is implicated in pre-mRNA splicing in spermatogenic cells.

The RNA-Seq results showed that *Sympk* deletion affected the splicing patterns of thousands of genes in spermatogenic cells, many of which have critical functions during spermatogenesis. As one of the top 10 most changed meiotic genes for the AS pattern, *Sun1* is a component of LINC complexes that mediates nuclear envelop attachment of meiotic telomeres and is also essential for driving chromosome synapsis and recombination (Scherthan et al., 1996; Ding et al., 2007; Chi et al., 2009; Koszul and Kleckner, 2009). BRDT, a testis-specific chromatin protein, specifically binds to acetylated histones and plays a key role in spermatogenesis (Pivot-Pajot et al., 2003; Manterola et al., 2018). *Meioc* is a conserved germ cell-specific gene expressed during male meiotic prophase I. MEIOC promotes the meiotic cell cycle program in coordination with YTHDC2 (Soh et al., 2017). The above genes were proven to be spliced alternatively in *Sympk* cKO spermatogenic cells. RANBP9 is a multifunctional protein in somatic cells and plays a novel role in regulating splicing in spermatogenic cells (Bao et al., 2014). Here, the splicing pattern of *Ranbp9* was changed and was verified by RT-PCR, indicating that the AS defects of many critical genes for germ cell development possibly resulted from the abnormal splicing of AS regulators, such as *Ranbp9*. In addition, the expression of *Sun1* was significantly reduced in *Sympk* cKO spermatogenic cells. Considering the phenotype of *Sun1*^–/–^ spermatocytes and the significance of *Sun1* in meiosis, we speculated that the meiotic defects of *Sympk*-deficient spermatocytes may be from the partial loss of SUN1. Additionally, 10 genes were upregulated and another 72 genes were downregulated in meiosis in the absence of SYMPK, but we do not know whether the phenotype was resulted by one or several of these misregulated genes.

In summary, our study demonstrates the requirement of SYMPK for meiosis and fertility in male mice and defines the role of SYMPK in the AS of numerous mRNAs required for normal spermatogenesis. However, more molecular details need to be delineated in the future, such as the role of SYMPK in the nuclear and cytoplasm polyadenylation of mRNAs in germ cells.

## Materials and Methods

### Animals

All mice were maintained on a C57BL/6-ICR mixed background and housed in the pathogen-free animal facility of Wuhan University. All animal experiments were conducted in accordance with the animal protocols approved by the Institutional Animal Care and Use Committee of Wuhan University. *Sympk*^*F/+*^ mice were purchased from Wellcome Trust Sanger Institute in London. *Ddx4*-Cre transgenic mice were provided by Dr. Xiaoyan Huang from Nanjing Medical University. *Stra8*-Cre transgenic mice were kindly provided by Dr. Minghan Tong from the State Key Laboratory of Molecular Biology in Shanghai.

All mice were genotyped by tail DNA extraction followed by gene-specific PCR using the specific primers (Supplementary Table 1).

### Antibody Generation

To generate a polyclonal antibody against SYCP3, full-length SYCP3 fused with His-tag was cloned into the pET42-b vector and expressed in *Escherichia coli*. Recombinant SYCP3 was affinity purified with Ni-NTA resin and used to immunize rabbits and mouse. The anti-SYCP3 sera were stored at -80°C until use. Anti-SYCP1 (aa871–946), anti-DDX4 (aa541–728), anti-MLH1 (aa528–737), and anti-HORMAD1 (aa2–277) sera were produced in the same way. The primers used for the construction of recombinant expression plasmids are listed in Supplementary Table 1.

### Histological Analyses, Immunostaining, and Imaging

For histological evaluation, testes from the *wt* control and *Sympk* cKO mice were isolated and fixed in Bouin’s solution overnight at room temperature, embedded with paraffin, and sectioned at 5–8 μm. After dewaxing and hydration, the sections were stained with hematoxylin and eosin.

For immunostaining, the samples were fixed in 4% paraformaldehyde (PFA) overnight at 4°C, dehydrated, embedded, and then sectioned using Cryostat Microtome (Leica CM1950, Heidelberg, Germany). TUNEL assays were performed using the *In Situ* Cell Death Detection Kit (Roche, Indianapolis, IN, United States). Nuclear spread analysis of the spermatocytes was performed as previously described (Luo et al., 2013). The primary antibodies used in this study are listed in Supplementary Table 2. Dylight 488- or Dylight 594-conjugated secondary antibody (Proteintech, Rosemont, Il, United States) was used at 1:100 dilutions, respectively. Histological and immunostaining images were captured with the Axio Imager 2 microscope (Zeiss, Oberchoken, Germany).

### Enrichment of Spermatogenic Cells

Spermatogenic cells were enriched using a two-step enzymatic digestion process followed by a differential adhesion method as previously described, with some modifications (Liu et al., 2017). Briefly, the testes were decapsulated, then the seminiferous tubules were transferred into 10 ml Krebs buffer with 500 μl 10 mg/ml collagenase, and incubated at 37°C, 120 rpm, for 10 min with gentle shaking every 3–5 min to accelerate testis dissociation.

Then, the cell suspension was centrifuged at 600 × *g* for 5 min and the pellet was digested in 10 ml Krebs buffer with 30 mg trypsin and 3 μg DNase I (preventing cells from clumping) and incubated at 37°C with shaking at 120 rpm for 10 min to dissociate the seminiferous tubules into single cells. The suspension was neutralized with 5 ml Dulbecco’s modified Eagle’s medium (DMEM) supplemented with 10% fetal bovine serum (FBS) and filtered through a 100-μm mesh cell strainer. After centrifuging at 600 × *g* for 5 min, the cell pellet was suspended in 10 ml of the ES medium and seeded in a 10-cm culture dish. After 4–6 h of short-term incubation at 37°C, the floating and weakly adhering cells were collected and identified using Western blotting against PLZF, SYCP1, SYCP3, and SOX9.

### Co-immunoprecipitation Followed by Mass Spectrometry and Western Blotting

Co-immunoprecipitation was performed with the spermatogenetic cells of P16 testes from *wt* and *Sympk* cKO^stra8–Cre^ mice using the anti-SYMPK antibody. Briefly, the spermatogenetic cells were homogenized in 1 ml lysis buffer (50 mM Tris, pH 7.5, 150 mM NaCl, 1% NP-40, 0.5% sodium deoxycholate, and 1 mM DTT) with 1 × cocktail (Roche) as a proteinase inhibitor. The lysates were treated with 0.5 mg/ml RNase A and 500 U/ml DNase at room temperature for 1 h, then pre-cleared with protein A agarose beads for 2 h at 4°C. Afterward, the anti-SYMPK antibody was added into the sample and incubated on an orbital shaker overnight at 4°C, followed by incubating with protein A agarose beads for 4 h at 4°C. The protein–beads complex was washed with lysis buffer three times.

For mass spectrometry, the purified protein–beads complex, shipped in solidified carbon dioxide, was subjected to HPLC followed by high-resolution mass spectrometry (HRMS) at the National Facility for Protein Science in Shanghai (NFPS), Zhangjiang Lab, China.

For Western blotting, the pre-cleared samples from the spermatogenetic cells of P16 testes from *wt* mice were divided into two parts and incubated with the anti-SYMPK antibody and immunoglobulin G (IgG) overnight at 4°C. The following steps were the same as those in the previous description. The protein–beads complex was boiled in 1 × sodium dodecyl sulfate (SDS) loading buffer for 10 min. After centrifuging at 1,2000 × *g* for 10min at 4°C, the supernatant was directly used for Western blotting analysis.

### Western Blotting

Protein samples were prepared using RIPA lysis buffer (20mM Tris, pH8, 150mM NaCl, 1% NP-40, 0.1% sodium deoxycholate, 1mM DTT, and 2mM EDTA, 100 μg/ml) with 1 × protease inhibitor cocktail (Roche). Then, the protein samples were separated in a 10% SDS-PAGE gel and transferred onto polyvinylidene difluoride (PVDF) membranes. After blocking with 5% non-fat milk for 1 h at room temperature, the membranes were incubated with diluted primary antibodies at 4°C overnight. After three washes with TBST (Tris-buffered saline/Tween 20), the membranes were incubated with secondary antibodies conjugated with horseradish peroxidase (AS014; ABclonal, Woburn, MA, United States) at room temperature for 1 h. The signals were developed with the SuperSignal^TM^ West Pico PLUS Chemiluminescent Substrate (#34577; ThermoFisher, Waltham, MA, United States), detected with Amersham Imager 600 (GE Healthcare Life Sciences, Marlborough, MA, United States). Information on the primary antibodies is listed in Supplementary Table 2.

### RNA Extraction and RT-qPCR

Total RNA was extracted from the spermatogenic cells of *wt* or *Sympk* cKO mice using the RNAprep Pure Micro Kit (TIANGEN, Beijing, China). Then, 2.5 μg of total RNA from each sample was subjected to reverse transcription into first-strand cDNA using the RevertAid RT Reverse Transcription Kit (K1691; ThermoFisher). For qPCR reaction, 140 ng cDNA was loaded as template in every 20 μl reaction volume for each sample. Real-time RT-PCR was performed using FastStart Essential DNA Green Master (06402712001; Roche) on a CFX Connect Real-Time PCR Detection System (Bio-Rad, Hercules, CA, United States). Relative gene expression was analyzed based on the 2^–△△Ct^ method with *Gapdh* as the internal control.

The primers used to determine the number of target genes were designed in PubMed combined with the Primer Premier 5.0 software. Primer information is listed in Supplementary Table 1.

### RNA Sequencing

Spermatogenic cells were prepared from 18-day-old control and *Sympk* cKO^Stra8–Cre^ mice. Total RNA was extracted using the RNAprep Pure Cell/Bacteria Kit (TIANGEN) according to the manufacturer’s protocol. Then, RNA quality was determined with the 2100 Bioanalyzer (Agilent, Palo Alto, CA, United States) and quantified using the ND-2000 (NanoDrop Technologies, Wilmington, DE, United States). Only high-quality RNA samples (OD_260_/OD_280_ = 1.8–2.2, OD_260_/OD_230_ ≥ 2.0, RIN ≥ 7.5, 28S:18S ≥ 1.0) were used to construct the sequencing library.

The RNA-Seq transcriptome library was prepared following the TruSeqTM RNA sample preparation kit from Illumina (San Diego, CA, United States) using 5 μg of total RNA. Shortly, the mRNA was isolated according to the polyA selection method by oligo(dT) beads and then firstly fragmented by a fragmentation buffer. Secondly, double-strand cDNA was synthesized using a SuperScript double-stranded cDNA synthesis kit (Invitrogen, Carlsbad, CA, United States) with random hexamer primers (Illumina). Then, the synthesized cDNA was subjected to end repair, phosphorylation, and “A” base addition according to Illumina’s library construction protocol. The libraries were size selected for cDNA target fragments of 200–300 bp on 2% Low Range Ultra Agarose followed by PCR amplification using Phusion DNA polymerase (NEB, Ipswich, MA, United States) for 15 PCR cycles. After quantification by TBS380, the paired-end RNA-Seq library was sequenced with the Illumina HiSeq xten (read length, 2 × 150 bp).

### Bioinformatics Analysis

The raw paired-end reads were trimmed and quality controlled using SeqPrep^1^ and Sickle^2^, with default parameters. Then, the clean reads were separately aligned to the reference genome with orientation mode using TopHat^3^ software.

To identify the differentially expressed genes (DEGs) between two different samples, the expression level of each transcript was calculated according to the fragments per kilobase of exon per million mapped reads (FRKM) method. RSEM^4^ was used to quantify gene abundances. The R statistical package software EdgeR (Empirical analysis of Digital Gene Expression in R)^5^ was utilized for analysis of differential expression. GO functional enrichment analysis was carried out by Goatools^6^. All the alternative splice events that occurred in our sample were identified using replicate multivariate analysis of transcript splicing (rMATS)^7^. Analysis of differential expression splicing (DES) was also performed using rMATS software.

### Statistics

Statistical analysis was performed using GraphPad Prism 5 software. The results of the statistical analyses were subjected to a Student’s *t*-test: ^∗∗∗^*p* < 0.001. The values were presented as the mean ± SD.

## Data Availability Statement

The datasets presented in this study can be found in online repositories. The names of the repository/repositories and accession number(s) can be found in the article/Supplementary Material.

## Ethics Statement

The animal study was reviewed and approved by Wuhan University.

## Author Contributions

ML and RW designed the project. RW and JZ performed most of the experiments. BZ and XH offered the mouse and analyzed phenotypes. XZ, ZC, JL, CL, and RL analyzed the data and phenotypes. All authors participated in the manuscript preparation and approved the final manuscript.

## Conflict of Interest

The authors declare that the research was conducted in the absence of any commercial or financial relationships that could be construed as a potential conflict of interest.

## Publisher’s Note

All claims expressed in this article are solely those of the authors and do not necessarily represent those of their affiliated organizations, or those of the publisher, the editors and the reviewers. Any product that may be evaluated in this article, or claim that may be made by its manufacturer, is not guaranteed or endorsed by the publisher.

## Footnotes

1
https://github.com/jstjohn/SeqPrep

2
https://github.com/najoshi/sickle

3 http://tophat.cbcb.umd.edu/, version2.0.0.

4
http://deweylab.biostat.wisc.edu/rsem/

5
http://www.bioconductor.org/packages/2.12/bioc/html/edgeR.html

6
https://github.com/tanghaibao/Goatools

7
http://rnaseq-mats.sourceforge.net/

## Supplementary Material

Supplementary Figure 1 SYMPK is absent in germ cells of *Sympk* cKO mice. Immunostaining of SYMPK in the testes of *wt* control and *Sympk* cKO mice at P4 **(A)** and P13 **(B)**. Scale bar, 50 μm.

Supplementary Figure 2 Phenotype of male mice with *Sympk* deficiency at embryonic stage by Ddx4-Cre. **(A)** Morphological analysis of testes from adult *wt* and *Sympk* cKO^Ddx4–Cre^ mice. **(B)** Histology of *Sympk* cKO^Ddx4–Cre^ testes at P10 and P16. Scale bar, 50 μm. **(C)** Histology of testes and cauda epididymidis from adult *Sympk* cKO^Ddx4–Cre^ male mice. Scale bar, 100 μm.

Supplementary Figure 3 SYMPK is required for the survival of spermatogonia and spermatocytes. **(A)** Immunostaining of PLZF in control and *Sympk* cKO^Stra8–Cre^ testes at P13. Scale bar, 50 μm. **(B)** Quantification of PLZF positive cells per seminiferous tubules in control and *Sympk* cKO^Stra8–Cre^ testes at P13 (*n* = 30 for control group, *n* = 69 for *Sympk* cKO^Stra8–Cre^ group). **(C)** Immunostaining of SYCP3 in control and *Sympk* cKO^Stra8–Cre^ testes at P13. Scale bar, 50 μm. **(D)** The number of SYCP3 positive cells in each *Sympk* cKO^Stra8–Cre^ tubule which containing SYCP3 positive cell and control tubule were counted, and compared between two groups. (*n* = 20 for both control group *Sympk* cKO^Stra8–Cre^ group). **(E)** Immunostaining of DDX4 in *wt* control and *Sympk* cKO^Ddx4–Cre^ testes at P4. Scale bar, 100 μm. **(F)** The number of DDX4 positive cells per seminiferous tubules in control and *Sympk* cKO^Ddx4–Cre^ tests at P4. At least 100 tubules were counted from two mice for each immunostaining assay. **(G)** Immunostaining of DDX4 in control, *Sympk* cKO^Ddx4–Cre^ and *Sympk* cKO^Stra8–Cre^ testes at P18. Scale bar, 50 μm. All sections were stained with DAPI to label nucleus.

Supplementary Figure 4 The localization of BRCA1 in *Sympk* cKO^Stra8–Cre^ spermatocytes. Immunostaining of meiotic chromosome spreads using the antibody against BRCA1. Scale bar, 20 μm.

Supplementary Figure 5 *Sympk* cKO^Stra8–Cre^ spermatocytes show reduced crossover formation. **(A)** Immunostaining for SYCP3 (red) and MLH1 (green) in control and *Sympk* cKO^Stra8–Cre^ spermatocytes. **(B)** Quantification of average MLH1 foci per spermatocyte. *Sympk*-deficient spermatocytes possess significantly fewer total MLH1 foci than wild-type spermatocyte (*n* = 17 for *wt*, *n* = 11for *Sympk* cKO). Scale bar, 20 μm.

Supplementary Figure 6 Identification of the enriched spermatogenic cells from testes by western blotting. Protein extracts from testes and enriched spermatogenic cells were analyzed by western blotting, using antibodies against PLZF, SYCP1, SYCP3, SOX9. PLZF are marker for spematogonia, SYCP1 and SYCP3 are markers for spermatocyte, SOX9 is a marker for Sertoli cell (Gao et al., 2006), and GAPDH was used as the loading control.
