# Supplementary material for: SYMPK Is Required for Meiosis and Involved in Alternative Splicing in Male Germ Cells
Source: Front Cell Dev Biol. 2021 Aug 9;9:715733. doi: 10.3389/fcell.2021.715733 (PMC8380814; doi:10.3389/fcell.2021.715733)
Supplement: Supplementary file 1 [file Data_Sheet_1.docx]

| **Supplementary Table 1. Primer list** | |  |
| --- | --- | --- |
| **Primer** | **Sequence(5’- 3’)** | **Application** |
| Sympk-F(P1) | GAGCTGTCTCCCCACTCCTGC | Genotyping |
| Sympk-R(P2) | TAAGGCCCACAGCTGGCTCGG |  |
| Sympk-ko-R(P3) | AATGCATACTGCTCTGGAAGACAC |  |
| Ddx4-F | CACGTGCAGCCGTTTAAGCCGCGT |  |
| Ddx4-R | TTCCCATTCTAAACAACACCCTGAA |  |
| Stra8-F | ACTCCAAGCACTGGGCAGAA |  |
| Stra8-R | GCCACCATAGCAGCATCAAA |  |
| SYCP1-U | GGAATTCCATATGCTTTTGAGCTTGGTTTCAGAGG | recombinant expression  plasmids construction |
| SYCP1-D | CAGTCTCGAGTTAAGTAAATAACTTTTCTGCTTCC |  |
| SYCP3-U | TCAATTCCATATGATGCTTCGAGGGTGTGGGGAC |  |
| SYCP3-D | GACGTGCCTGCAGGAATAACATGGATTGAAGAGAC |  |
| DDX4-U | CTCCATATGAGAACTATGGTCTTTGTTGAAACCAAG |  |
| DDX4-D | AGAATCTCGAGATCCCATGACTCGTCATCAAC |  |
| MLH1-U | ACGCGGTCATATGATGCTCCGTAACCATTCCTTTG |  |
| MLH1-D | GCTTCTCGAGATGCTTCGGAGGTAGGAGGTGTG |  |
| HORMAD1-U | GGGAAACCAAGCTTACTATGCAGTTGCAGAGGAC |  |
| HORMAD1-D | CCGGGAGGCTCGAGCTGTTCGTTCATTTTAGTTT |  |
| Sun1-U | CCTGATTCGTGAGCAGACCAAAGTG | determination of  splicing defects |
| Sun1-D | AGTTGCGAGTGGGTGGCTGTGTGTA |  |
| Ranbp9-U | CCGGGAAGCGCAGGAGGAAGCA |  |
| Ranbp9-D | CACCCGCAGGTTGTTCTGCGAGAGG |  |
| Brdt -U | CTACCATTGCTACAGAGGCTCG |  |
| Brdt -D | GCTTCATTATGTGTGTCTTGGTC |  |
| Meioc-U | CTGGGCCTCGACGTCCTCCCTCAC |  |
| Meioc-D | GAGACTTGTAGCAATCATAGAAGGGCGCCG |  |
| Stra8-U | CAAAGTGGCAGGTACTGAATAGGAC | RT-qPCR |
| Stra8-D | TTGACCTCCTCTAAGCTGTTGGG |  |
| Meioc-U | AGCCAATGGCACACCTGAGAC |  |
| Meioc-D | TGCTGCTTCCTTTGCAAACTG |  |
| Brdt-U | ATCGAGATCGACTTTGAGACCCTG |  |
| Brdt-D | CTCCTCCTTAGACCTGACAACCTTC |  |
| DMC1-U | CAGTACAGTGAAAGGAGGAAGATGG |  |
| DMC1-D | CACCACAGAGGGTATGAGACAGC |  |
| Sun1-U | CGGAGAACACTGGCTACACTTACG |  |
| Sun1-D | TGCTGGTGCTAATGTGCGAATC |  |
| Ythdc2-U | AGAAACAACAGACCACCCTCACAG |  |
| Ythdc2-D | GAAGACAGCATCACCTCCATCATC |  |
| Atrx-U | CGTTTCAACATACCAACTGGGAC |  |
| Atrx-D | CCGTCATACTGTTCGTGGCTTC |  |
| Dnmt3a-U | AGGGAAAGATCATGTACGTCGG |  |
| Dnmt3a-D | CAGGAGGCGGTAGAACTCAAAG |  |
| Gapdh-U | CCCCAATGTGTCCGTCGTG |  |
| Gapdh-D | TGCCTGCTTCACCACCTTCT |  |

| **Supplementary Table 2. Primary antibody list** | | |
| --- | --- | --- |
| **Antibody** | **Resource** | **Usage and dilution** |
| Anti-γH2AX | 05-636, Merck | IF 1:200 |
| Anti-PLZF | A5863, ABclonal | WB 1:1000 |
|  | sc-28319, Santa Cruz | IF 1:100 |
| Anti-GAPDH | 10494-1-AP, Proteintech | WB 1:2000 |
| Anti-BRCA1 | Gift from Dr. LinYu Lu, Zhejiang university | IF 1:200 |
| Anti- RPA2 | Generated by our lab | IF 1:100 |
| Anti- MEIOB | Generated by our lab | IF 1:100 |
| Anti- DMC1 | Gift from Dr. QingHua Shi | IF 1:50 |
| Anti-RAD51 | PC-130, Merck | IF 1:25 |
| Anti- MLH1 | Generated by our lab | IF 1:25 |
| Anti- SYCP1 | Generated by our lab | WB 1:100 |
|  |  | IF 1:100 |
| Anti-SYCP3 | Generated by our lab | WB 1:100 |
|  |  | IF 1:100 |
| Anti-HORMAD1 | Generated by our lab | IF 1:100 |
| Anti-SYMPK | 11519-1-AP, Proteintech | IF 1:100 |
|  |  | WB 1:500 |
| Anti-SOX9 | A19710, ABclonal | WB 1:2000 |
| Anti-DDX4 | generated by our lab | IF 1:100 |
| Cy3-PNA | Vector Labs | IF 1:200 |
| Anti-SUN1 | A16024, ABclonal | WB 1:1000 |
| Anti-PRPF8 | A6053, ABclonal | IF 1:100 |
|  |  | WB 1:1000 |
| Anti-DDX5 | A11339, ABclonal | IF 1:100 |
|  |  | WB 1:1000 |

**Supplementary Table 3. Proteins associated with pre-mRNA processing detected in IP-MS**

| Proteins | *wt* | | *Sympk* cKO | |
| --- | --- | --- | --- | --- |
|  | Peptides | Unique | Peptides | Unique |
| PRPF8 | 47 | 46 | 4 | 3 |
| DDX5 | 26 | 17 | 16 | 11 |
| PRPF40a | 20 | 19 | 0 | 0 |
| DHX15 | 19 | 12 | 5 | 2 |
| WTAP | 16 | 15 | 9 | 8 |
| PRPF38b | 12 | 11 | 1 | 1 |
| PRPF19 | 7 | 5 | 0 | 0 |
| CWC22 | 6 | 6 | 0 | 0 |
