# Supplementary figures and images for: SYMPK Is Required for Meiosis and Involved in Alternative Splicing in Male Germ Cells
Source: Front Cell Dev Biol. 2021 Aug 9;9:715733. doi: 10.3389/fcell.2021.715733 (PMC8380814; doi:10.3389/fcell.2021.715733)

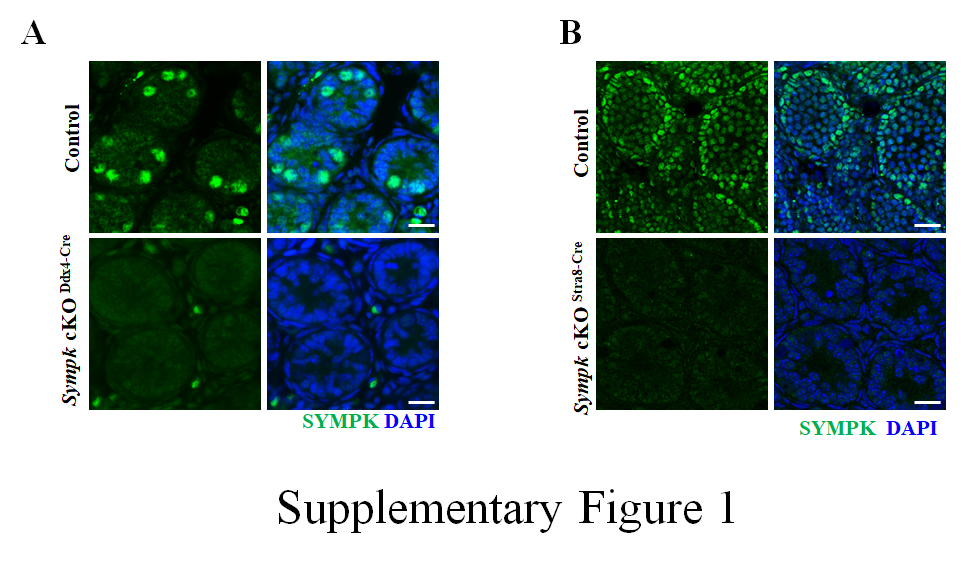

Supplement: Supplementary Figure 1 — SYMPK is absent in germ cells of Sympk cKO mice. Immunostaining of SYMPK in the testes of wt control and Sympk cKO mice at P4 (A) and P13 (B). Scale bar, 50 μm. [file Image_1.tif]

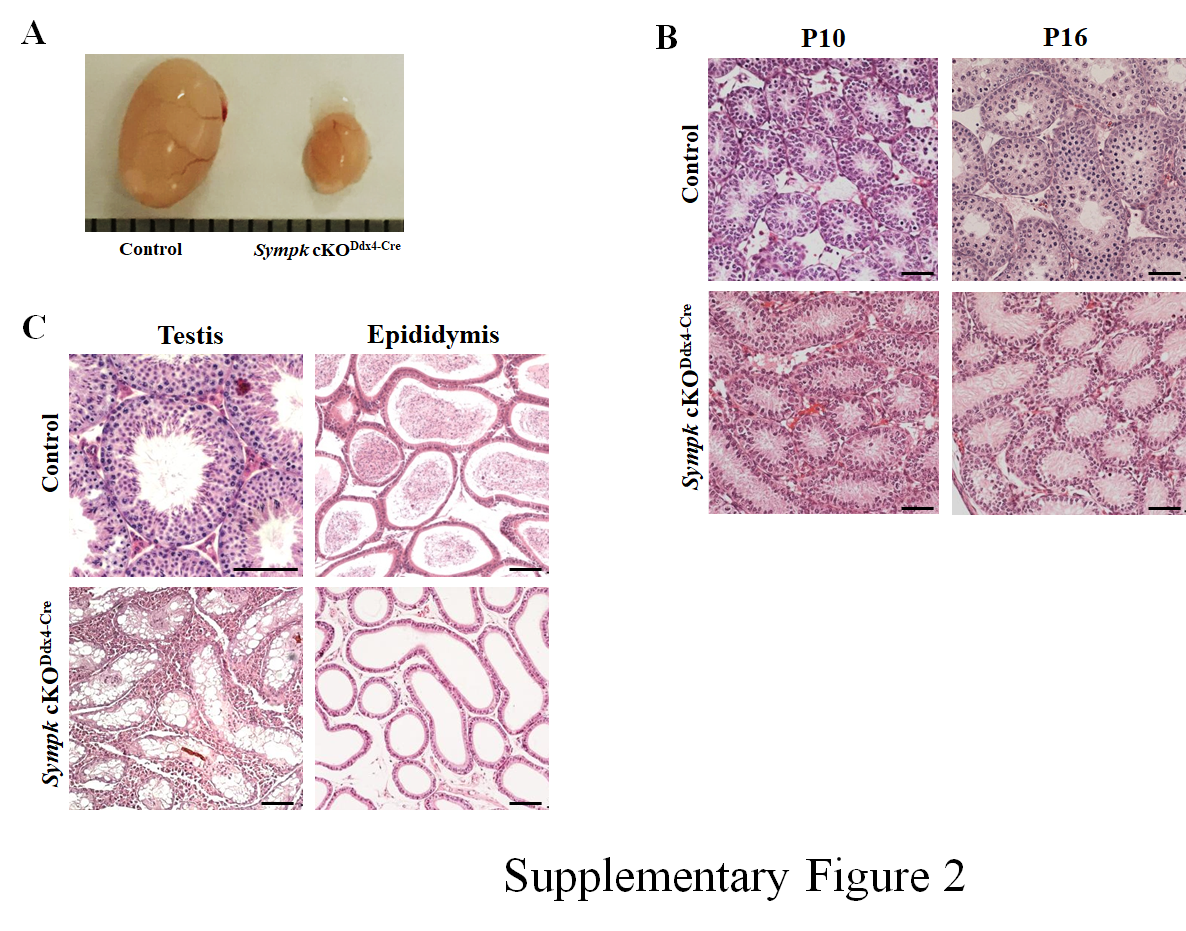

Supplement: Supplementary Figure 2 — Phenotype of male mice with Sympk deficiency at embryonic stage by Ddx4-Cre. (A) Morphological analysis of testes from adult wt and Sympk cKODdx4–Cre mice. (B) Histology of Sympk cKODdx4–Cre testes at P10 and P16. Scale bar, 50 μm. (C) Histology of testes and cauda epididymidis from adult Sympk cKODdx4–Cre male mice. Scale bar, 100 μm. [file Image_2.tif]

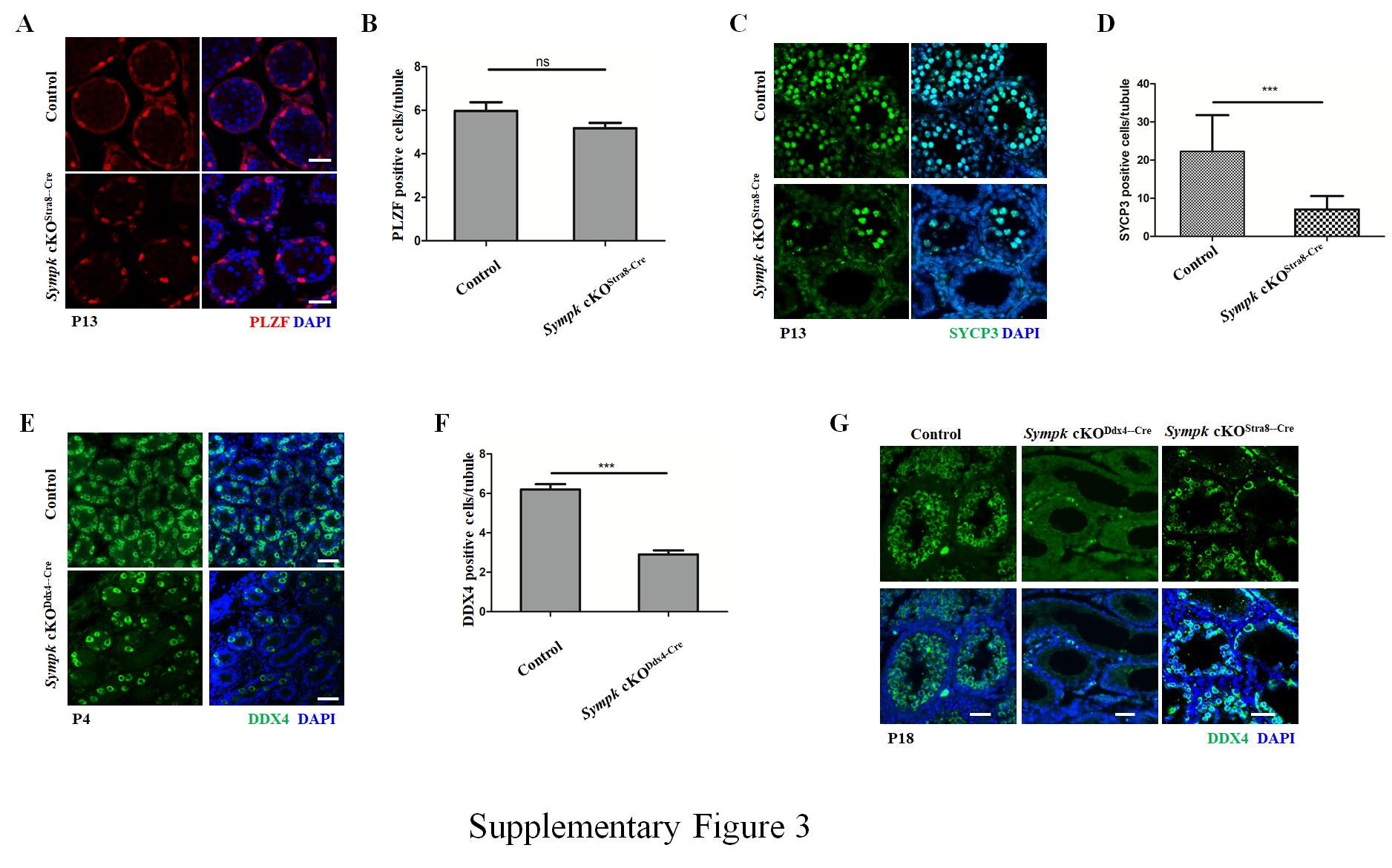

Supplement: Supplementary Figure 3 — SYMPK is required for the survival of spermatogonia and spermatocytes. (A) Immunostaining of PLZF in control and Sympk cKOStra8–Cre testes at P13. Scale bar, 50 μm. (B) Quantification of PLZF positive cells per seminiferous tubules in control and Sympk cKOStra8–Cre testes at P13 (n = 30 for control group, n = 69 for Sympk cKOStra8–Cre group). (C) Immunostaining of SYCP3 in control and Sympk cKOStra8–Cre testes at P13. Scale bar, 50 μm. (D) The number of SYCP3 positive cells in each Sympk cKOStra8–Cre tubule which containing SYCP3 positive cell and control tubule were counted, and compared between two groups. (n = 20 for both control group Sympk cKOStra8–Cre group). (E) Immunostaining of DDX4 in wt control and Sympk cKODdx4–Cre testes at P4. Scale bar, 100 μm. (F) The number of DDX4 positive cells per seminiferous tubules in control and Sympk cKODdx4–Cre tests at P4. At least 100 tubules were counted from two mice for each immunostaining assay. (G) Immunostaining of DDX4 in control, Sympk cKODdx4–Cre and Sympk cKOStra8–Cre testes at P18. Scale bar, 50 μm. All sections were stained with DAPI to label nucleus. [file Image_3.tif]

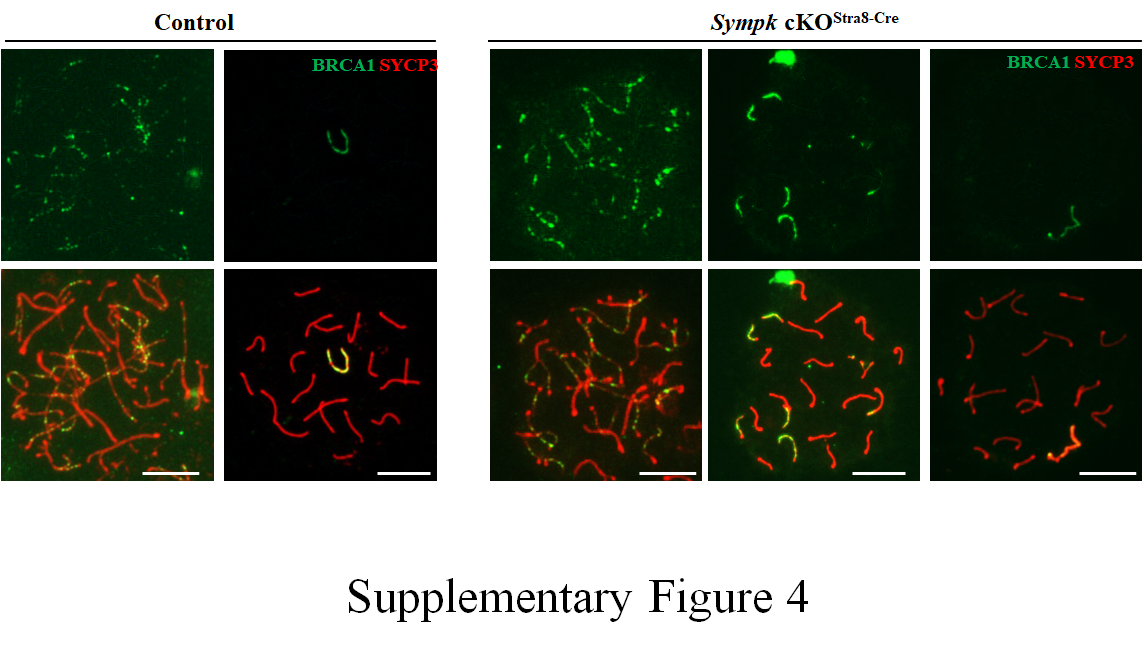

Supplement: Supplementary Figure 4 — The localization of BRCA1 in Sympk cKOStra8–Cre spermatocytes. Immunostaining of meiotic chromosome spreads using the antibody against BRCA1. Scale bar, 20 μm. [file Image_4.tif]

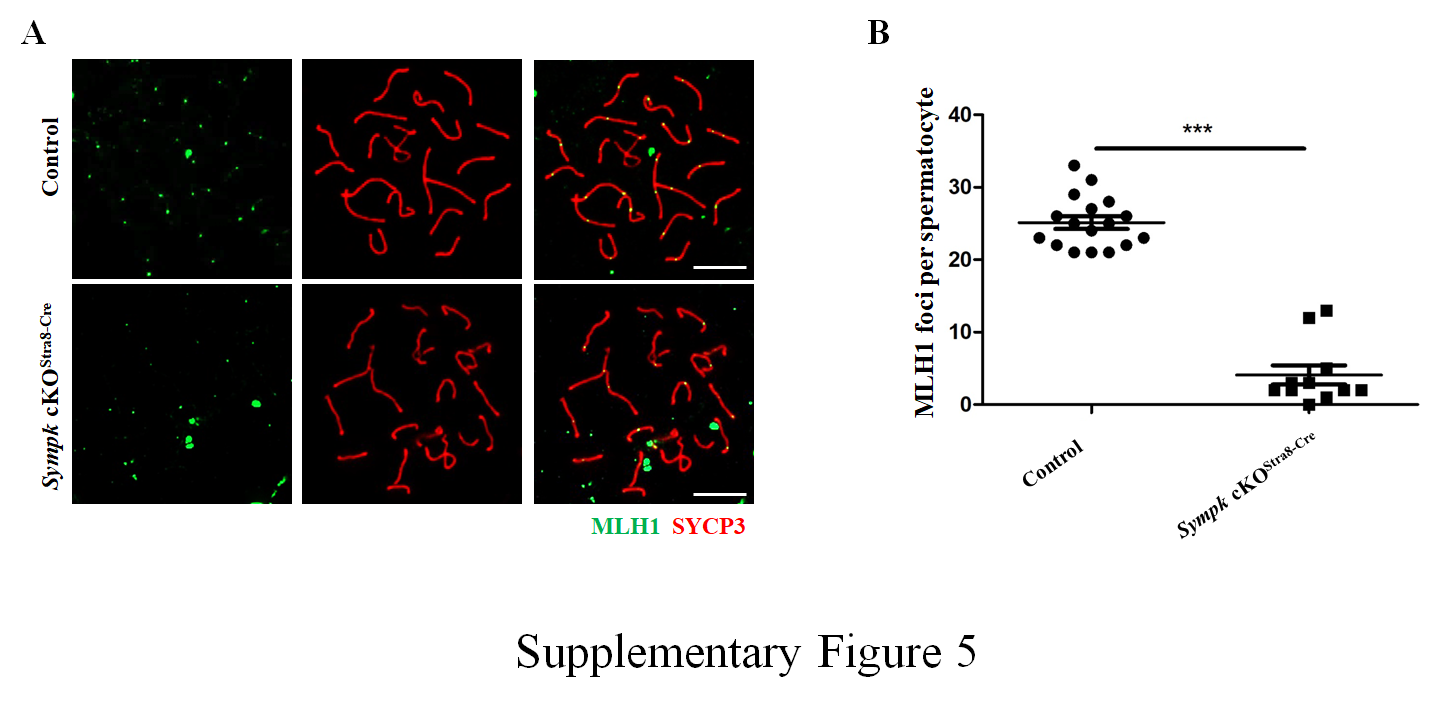

Supplement: Supplementary Figure 5 — Sympk cKOStra8–Cre spermatocytes show reduced crossover formation. (A) Immunostaining for SYCP3 (red) and MLH1 (green) in control and Sympk cKOStra8–Cre spermatocytes. (B) Quantification of average MLH1 foci per spermatocyte. Sympk-deficient spermatocytes possess significantly fewer total MLH1 foci than wild-type spermatocyte (n = 17 for wt, n = 11for Sympk cKO). Scale bar, 20 μm. [file Image_5.tif]

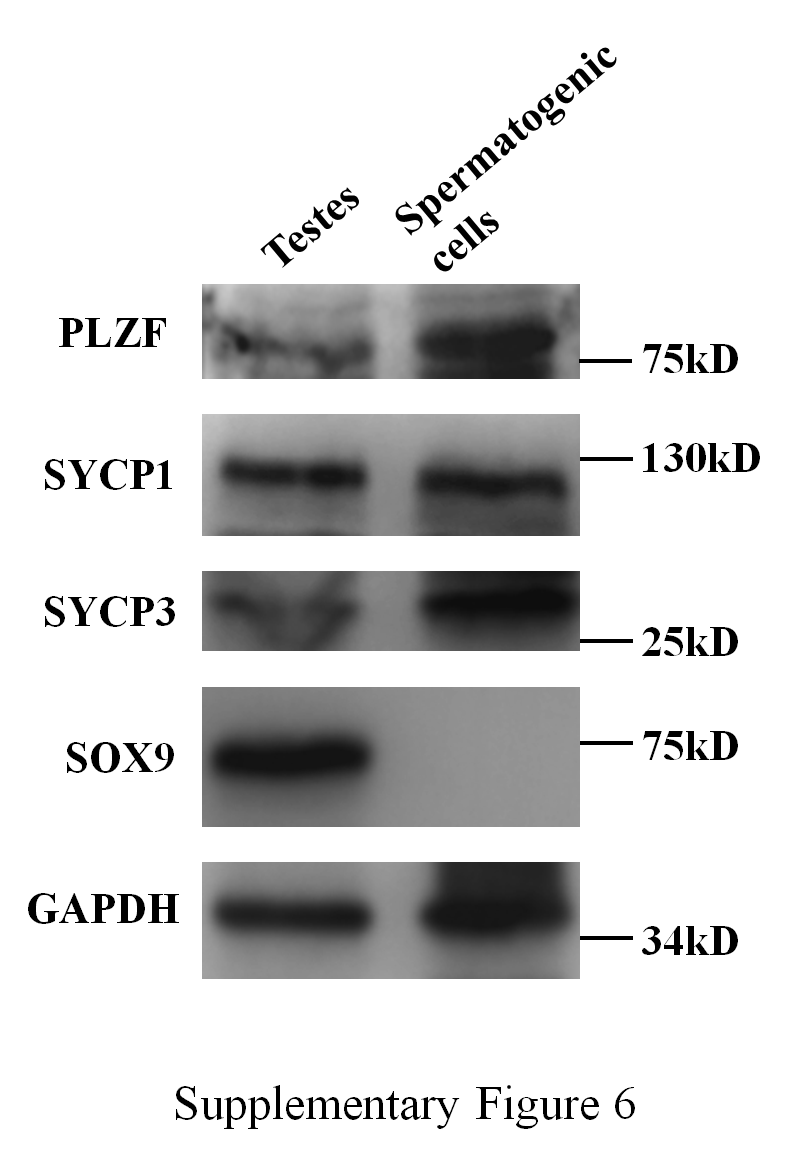

Supplement: Supplementary Figure 6 — Identification of the enriched spermatogenic cells from testes by western blotting. Protein extracts from testes and enriched spermatogenic cells were analyzed by western blotting, using antibodies against PLZF, SYCP1, SYCP3, SOX9. PLZF are marker for spematogonia, SYCP1 and SYCP3 are markers for spermatocyte, SOX9 is a marker for Sertoli cell (Gao et al., 2006), and GAPDH was used as the loading control. [file Image_6.tif]
